# Multi‐Instrument Characterization of Magnetospheric Cold Plasma Dynamics in the June 22, 2015 Geomagnetic Storm

**DOI:** 10.1029/2021JA029292

**Published:** 2021-06-21

**Authors:** M. Vellante, K. Takahashi, A. Del Corpo, I. S. Zhelavskaya, J. Goldstein, I. R. Mann, E. Pietropaolo, J. Reda, B. Heilig

**Affiliations:** ^1^ Department of Physical and Chemical Sciences University of L'Aquila L'Aquila Italy; ^2^ The Johns Hopkins University Applied Physics Laboratory Laurel MD USA; ^3^ Helmholtz Centre Potsdam GFZ German Research Centre for Geosciences and University of Potsdam Potsdam Germany; ^4^ Space Science and Engineering Division Southwest Research Institute San Antonio TX USA; ^5^ University of Alberta Edmonton AB Canada; ^6^ Institute of Geophysics Polish Academy of Sciences Warsaw Poland; ^7^ Mining and Geological Survey of Hungary Budapest Hungary; ^8^ Eötvös Loránd University Budapest Hungary

**Keywords:** Field line resonance, ground‐based magnetometers, magnetoseismology, plasmasphere, Swarm satellites, Van Allen Probes

## Abstract

We present a comparison of magnetospheric plasma mass/electron density observations during an 11‐day interval which includes the geomagnetic storm of June 22, 2015. For this study we used: Equatorial plasma mass density derived from geomagnetic field line resonances (FLRs) detected by Van Allen Probes and at the ground‐based magnetometer networks EMMA and CARISMA; in situ electron density inferred by the Neural‐network‐based Upper hybrid Resonance Determination algorithm applied to plasma wave Van Allen Probes measurements. The combined observations at *L* ∼ 4, MLT ∼ 16 of the two longitudinally separated magnetometer networks show a temporal pattern very similar to that of the in situ observations: A density decrease by an order of magnitude about 1 day after the Dst minimum, a partial recovery a few hours later, and a new strong decrease soon after. The observations are consistent with the position of the measurement points with respect to the plasmasphere boundary as derived by a plasmapause test particle simulation. A comparison between plasma mass densities derived from ground and in situ FLR observations during favorable conjunctions shows a good agreement. We find however, for *L* < ∼3, the spacecraft measurements to be higher than the corresponding ground observations with increasing deviation with decreasing *L*, which might be related to the rapid outbound spacecraft motion in that region. A statistical analysis of the average ion mass using simultaneous spacecraft measurements of mass and electron density indicates values close to 1 amu in plasmasphere and higher values (∼2–3 amu) in plasmatrough.

## Key Points

- The combination of longitudinally separated magnetometer arrays reproduces main magnetospheric plasma density variations observed in situ
- Observations are consistent with predictions provided by a plasmapause test particle simulation
- Plasma mass densities derived from ground and in situ field line resonance (FLR) observations for L > 3 are in good agreement during favorable conjunction intervals

## 1 Introduction

Understanding the concentration and composition of the plasma populating the Earth's magnetosphere, its spatial distribution and its temporal variations, represent relevant information in the space weather context. First of all, the mass density determines the inertia of the plasma and consequently the magnetohydrodynamic (MHD) response of the magnetosphere to solar wind perturbations. It also determines the frequency of Ultra‐Low‐Frequency (ULF) waves which can energize radiation belt particles (e.g., Elkington et al., 1999). Also, the ion composition affects the growth and evolution of electromagnetic ion cyclotron (EMIC) waves (e.g., Denton et al., 2014) which are an important loss mechanism for radiation belt electrons (e.g., Shprits et al., 2008). Another important aspect in the space weather context is the contribution of the plasmasphere (the region of cold and dense plasma encircling the Earth and approximately corotating with it) to the Total Electron Content (TEC) of the ionosphere. Plasmasphere density variations may then cause Global Navigation Satellite System (GNSS) inaccuracies and communications problems (Jakowski & Hoque, 2018).

The most dramatic changes in plasma density and in its spatial distribution occur during geomagnetic storms. In particular, during events of southward turning of the interplanetary magnetic field (IMF), the enhanced dawn‐dusk convection electric field may significantly erode the nightside plasmasphere (e.g., Goldstein et al., 2003), bringing its boundary (the plasmapause) from the typical distance of 4–5 Earth radii (R_E_) up to ∼2 R_E_ for the most extreme events (e.g., Chi et al., 2000). At the same time, the dayside plasmasphere moves sunward to form a broad plume which becomes progressively narrower. As the convection electric field decreases, the new nightside plasmasphere boundary corotates into the dayside sector and the plume also starts rotating with the Earth (e.g., Goldstein et al., 2005). The plasmaspheric material stripped away by this process convects through the dayside magnetosphere and is lost to the magnetopause. As a result of this, some magnetospheric regions which were previously filled by the dense plasmaspheric material may become part of the much less dense plasmatrough region with density decreases by a factor of up to two orders of magnitude (Carpenter & Anderson, 1992). During the recovery phase of the geomagnetic storm, the depleted flux tubes are refilled by the ionosphere on a much longer time scale (of the order of days, e.g., Chi et al., 2000; Obana et al., 2010; Park, 1974). Another storm time effect is an enhancement of the oxygen ion population in the vicinity of the plasmapause during the initial and recovery phase of the storm (e.g., Fraser et al., 2005; Horwitz et al., 1984; Nosé et al., 2015).

Electron and mass density of the magnetospheric plasma can be measured by different techniques both in space and from the ground. The electron number density can be measured locally by plasma wave experiments on board of satellites (e.g., Décréau et al., 1997; Kurth et al., 2015) and can also be derived from the spacecraft potential (e.g., Escoubet et al., 1997; Jahn et al., 2020). From the ground, it can be measured by detection of Very Low Frequency (VLF) whistlers propagating along the geomagnetic field lines (e.g., Park, 1972). On the other hand, the plasma mass density can be inferred from satellite (Takahashi et al., 2006) and ground (Menk & Waters, 2013) detection of geomagnetic field line resonances (FLR). In situ measurements of the concentration of different ions have been also reported (e.g., Horwitz et al., 1984; Sandhu et al., 2016), but spacecraft charging effects often prevent the detection of the ions in the low energy range (Moldwin, 1997).

Each of these measurements can provide information at a given time only at particular points in space and therefore, taken alone, provide only a very limited description of the dynamic processes occurring in the magnetosphere, especially along the world‐line of individual satellites. Of course, in the absence of available in‐situ satellite plasma measurements the ability to remote sense mass dynamics from the ground becomes of increased importance.

Global images of the plasmasphere (in terms of helium contribution) have been provided in the past from the Extreme UltraViolet (EUV) Imager on the Imager for Magnetopause‐to‐Aurora Global Exploration (IMAGE) satellite (Sandel et al., 2000). These images could be also converted to He^+^ density maps in the equatorial plane (Sandel et al., 2003) allowing a quantitative comparison with other typical density measurements, but for conversion into total mass density it requires assumptions of the relative abundance of He^+^, H^+^ and other species. These global images revealed a lot of detailed structures in the plasmasphere (plumes, notches, channels, shoulders, etc.,) which could be followed in their initial formation and time evolution in response to the variable conditions of the solar wind (Spasojević et al., 2003). No similar experiments are presently in operation (the IMAGE mission was operative from 2000 to 2005). It is therefore very important when investigating the dynamics of the magnetospheric plasma (for example during a geomagnetic storm) to combine as many measurements as possible at different locations and from different instruments/techniques to get a more complete picture of the ongoing processes. This is also important for the intercalibration of the different techniques.

It is also extremely useful for the interpretation of local variations to compare the observations with predictions provided by models and simulations. In this regard, the plasmapause test particle (PTP) simulation has been proven to be very effective, providing the global shape of the plasmapause at any given time using an ensemble of cold test particles subject to ExB drift (Goldstein et al., 2005; Goldstein, Thomsen, & DeJong, 2014; Goldstein, De Pascuale, et al., 2014).

Some coordinated ground‐based and satellite observations have been conducted in the past (Carpenter et al., 1981; Clilverd et al., 2003; Dent et al., 2003, 2006; Grew et al., 2007; Maeda et al., 2009). They included different combinations of ground‐based FLR measurements, ground‐based whistler measurements, in situ electron and He^+^ density measurements. These studies showed a good consistency among the different measurements and enabled in some cases to infer the concentration and the dynamics of the heavy ions during different geomagnetic activity conditions. Combined measurements of electron and mass density have been also conducted by using only in situ measurements from the same satellite (Nosé et al., 2011, 2015; Takahashi et al., 2006, 2008). These studies confirmed for example the formation of an oxygen torus near the plasmapause during the initial and recovery phase of a geomagnetic storm (Fraser et al., 2005).

In this paper we present a comparative study of plasma mass/electron density observations during an 11‐day interval which includes the geomagnetic storm of June 22, 2015 (Dst minimum of −204 nT). The data used for this study are: (a) equatorial plasma mass density derived from FLRs detected using the ground‐based magnetometer networks European quasi‐Meridional Magnetometer Array (EMMA, Lichtenberger et al., 2013) and Canadian Array for Realtime InvestigationS of Magnetic Activity (CARISMA, Mann et al., 2008); (b) equatorial/local plasma mass density derived from FLRs detected by Van Allen Probes (Takahashi et al., 2015); (c) in situ electron number density measurements by Van Allen Probes (Neural‐network‐based Upper hybrid Resonance Determination (NURD) data, Zhelavskaya et al., 2016). Measurements are also compared with the expected temporal evolution of the plasmapause shape from plasmapause test particle simulations (Goldstein, De Pascuale, et al., 2014).

The remainder of the paper is organized as follows. Section 2 presents an overview of the examined interval and describes the experiments, data and methods used for the analysis. Section 3 presents a comparative study among the different kind of observations. Section 4 presents conclusions.

## 2 Data and Method

### 2.1 Ground Measurements

The event under study (June 18–28, 2015) was already examined in a previous paper (Piersanti et al., 2017) but using only plasma mass density estimates derived from EMMA‐FLR observations. The adopted technique is comprehensively described in Del Corpo et al. (2019, 2020). Briefly, fundamental FLR frequencies were evaluated for the mid‐point of 37 pairs of stations slightly separated in latitude (1–3°) using the cross‐phase technique (Waters et al., 1991). Typical uncertainties in the frequency estimates are of the order of ∼15%, as resulting from the bandwidth of the cross‐phase peak and/or from the difference between the frequency derived using the cross‐phase method and the frequency derived using the power ratio method (e.g., Berube et al., 2003; Del Corpo et al., 2019). Each frequency, determined with a time step of half an hour, was then converted to the equatorial plasma mass density *ρ*_eq_ by solving the MHD wave equation for the toroidal mode (Singer et al., 1981). This was done at each time step by using the T02 Tsyganenko magnetic field model (Tsyganenko, 2002) and a radial dependence of the density along the field lines as given by the power law model:

(1) $$\rho =\rho_{eq}{\left(\frac{r_{eq}}{r}\right)}^{m},$$

where *r* is the geocentric distance, and *r*_eq_ is the equatorial distance. Following indications from previous studies (e.g., Denton et al., 2006; Takahashi & Denton, 2021; Takahashi et al., 2004; Vellante & Förster, 2006), we found it appropriate to use a power law index *m* = 1. The effect of using different power law indices is discussed in Section 3.2. The uncertainty in the calculated mass densities related to the uncertainty in the estimated FLR frequency is of the order of 30%, as *ρ*_eq_ ∝ 1/*f* ^2^. Equatorial densities were then evaluated at any given distance in the local time sector monitored by EMMA by applying a smoothing spline to the radial profiles (Del Corpo et al., 2019).

As is well known theoretically (e.g., Hughes & Southwood, 1976) and experimentally (e.g., Chi et al., 2013; Del Corpo et al., 2019; Wharton et al., 2019), at nighttime the low ionospheric conductivity generally prevents the formation of FLRs and therefore the method was usable only during daytime hours.

**Figure 1:**
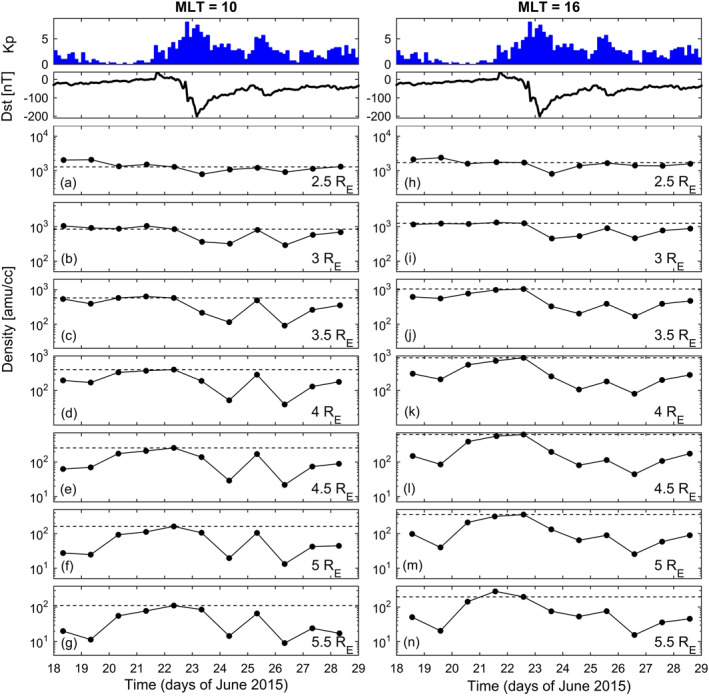
(a–n) Day‐to‐day variation of the equatorial plasma mass density at different geocentric distances and for two different Magnetic Local Times, derived from field line resonance frequencies detected at the EMMA magnetometer network. Dashed horizontal lines in each panel indicate the density level of June 22, when the storm effects are not yet evident. Top panels show the Kp and Dst indices.

An overview of the temporal variation of the mass density for the event on a daily scale is shown in Figure 1 along with the geomagnetic indices Kp and Dst. The observations refer to the equatorial distances: 2.5, 3.0, 3.5, 4.0, 4.5, 5.0, 5.5 R_E_ and two different magnetic local times (MLT): 10 and 16. Throughout the paper, we define the MLT of a given point in space as the longitude in the solar magnetic (SM) reference frame of the equatorial crossing point using field line mapping. The longitude ϕ is then converted in hours (MLT (h) = 12 + ϕ(deg)/15). Dashed horizontal lines in each panel indicate the density level of June 22 when the storm effects are not yet evident. In the morning sector (left panels) the density variation is characterized by a strong decrease on June 24 (1 day after the Dst minimum) for *r* ≥ 3 R_E_ (panels b–g), followed by an almost complete recovery on the next day, a new decrease (even stronger) on June 26, and a more gradual recovery on the next days. A similar pattern is observed on the afternoon sector but the recovery on June 25 was only partial.

**Figure 2:**
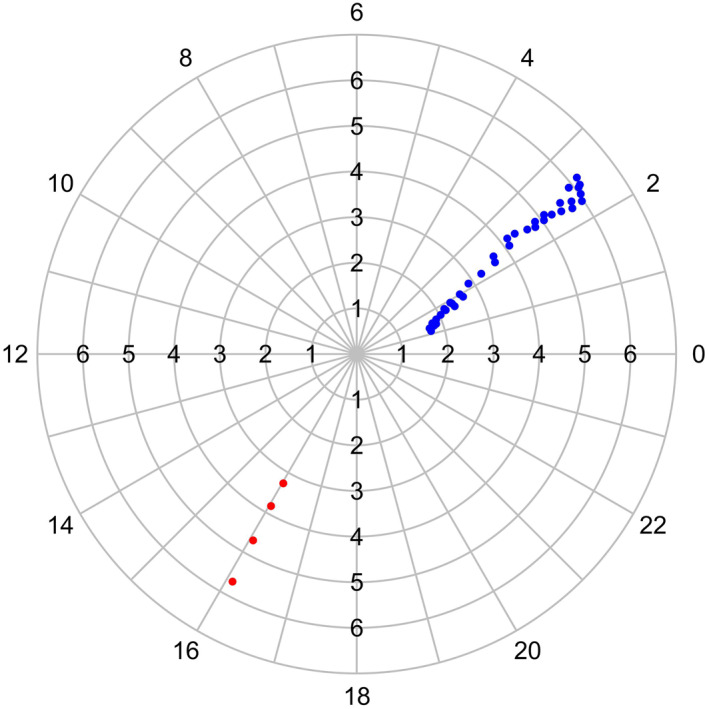
Locations of station pair midpoints for EMMA (blue dots) and CARISMA (red dots) in *L*‐MLT coordinates. Values reported around the outermost circle are the difference between MLT and UT. The mapping has been done using the IGRF model at 00 UT of June 21, 2015.

Using a single meridional array we can monitor a given MLT region only every 24 h. In order to increase the monitoring rate, we extended the analysis by using magnetometer data from the Alberta line (∼310° CGM longitude) of the CARISMA network (Mann et al., 2008; https://www.carisma.ca) which is longitudinally separated from EMMA by ∼150° (∼10 h in MLT). Station pairs which were available and useful for the present analysis are reported in Table 1. Figure 2 shows the mapping to the magnetic equatorial plane (using IGRF) of the EMMA (blue dots) and CARISMA (red dots) mid points of the station pairs used for the analysis. Longitude values, reported around the outermost circle in Figure 2, are expressed in terms of the difference between MLT and UT.

**Table 1:** CARISMA Station Pairs Employed^a^

Station pair	Geog. Lat. (°N)	Geog. Lon. (°E)	CGM Lat. (°N)	CGM Lon. (°E)	*L*	MLT	CGM Lat.Separ. (°)	CGM Lon.Separ. (°)
FCHP‐MCMU	57.72	248.84	64.95	310.69	5.68	UT – 7.91 h	2.06	0.54
MCMU‐MSTK	55.00	247.91	62.16	310.28	4.67	UT – 7.94 h	3.52	1.35
MSTK‐VULC	51.86	247.03	58.93	309.99	3.83	UT – 7.96 h	2.94	0.74
VULC‐POLS	49.02	246.41	56.02	309.90	3.26	UT – 7.97 h	2.90	0.88

^a^ *L* shell, geomagnetic coordinates, and MLT are calculated for June 21, 2015, 00:00 UT at 120 km altitude.

The mapping shown in Figure 2 is representative of quiet conditions. It can actually change significantly during disturbed conditions because of the effect of the magnetospheric currents. In particular, during periods of enhanced ring current, higher latitude stations may map to much larger equatorial distances (e.g., Berube et al., 2006; Del Corpo et al., 2020). Throughout the paper we actually used the field line mapping given by the T02 model which takes in due account the effect of the ring current. We also examined the effect of using the Tsyganenko TS05 model (Tsyganenko & Sitnov, 2005) which is considered to provide a better representation of the magnetospheric field during storm conditions. We found that the mapping to the equator could change significantly for the highest latitude stations during periods of high geomagnetic activity. Nevertheless, the estimated mass density at a given radial distance (as evaluated using a smoothing spline to the radial profiles) did not change significantly when using the T02 or the TS05 model, except for very highly disturbed times. In particular, we found that in the range *r*_eq_ ≤ 5 R_E_ (where our study is mostly focused on) the deviation between T02 and TS05 density estimates was typically less than 10% and only during the two most disturbed days of the present study reached ∼25%. This result made us more confident in the reliability of the comparison between ground based and in situ observations which will be presented in Section 3.

CARISMA data were processed for the whole period by applying the same technique used for the EMMA data. For the present study we restricted the analysis to *r*_eq_ = 4 R_E_ where the CARISMA data coverage was the best. As a matter of fact, this distance was generally very close to the equatorial region mapped by the Ministik Lake‐Vulcan (MSTK‐VULC) station pair (*L* = 3.83). It also corresponds to the mean equatorial radius of the plasmapause (Carpenter, 1968), and so it is a suitable location for monitoring the plasmasphere dynamics.

### 2.2 In Situ Measurements

**Figure 3:**
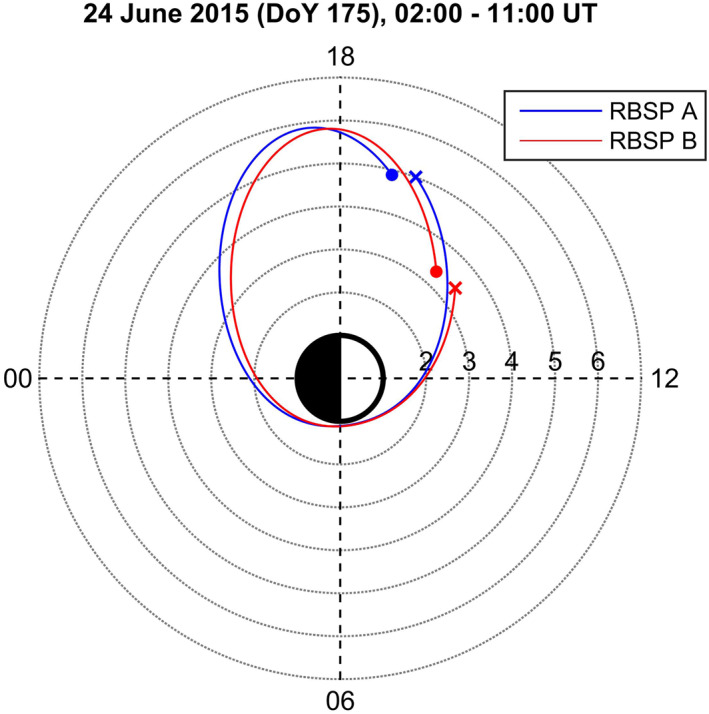
Radiation Belt Storm Probes (RBSP) orbits in *L*‐MLT coordinates (calculated using a centered dipole) during 0200–1100 UT on June 24, 2015. Locations at start/end times are indicated by dots/crosses.

During the investigated period the orbits of the Van Allen Probes, formerly known as Radiation Belt Storm Probes (RBSP, Mauk et al., 2013), were characterized by outbound and inbound legs occurring in the daytime and night‐time sectors, respectively. An example, for June 24, 2015, is shown in Figure 3 using *L*‐MLT coordinates. The blue/red line refers to RBSP A/RBSP B, respectively, with Probe A preceding Probe B by ∼1 h. In the morning sector the Van Allen Probes were very close to the Earth (*L* < 2), so only the orbit section occurring in the afternoon was usable for a comparison with the daytime ground observations. In particular, the spacecraft crossed the *L*‐shell = 4 at MLT ∼ 16. The orbit characteristics changed little with time during the investigated period, so each Van Allen Probe crossed the same *L*‐MLT region approximately every 9 h.

We used RBSP electron number density values as derived by applying the NURD algorithm (Zhelavskaya et al., 2016) to plasma wave measurements made by the High Frequency Receiver (HFR) instrument of the Electric and Magnetic Field Instrument Suite and Integrated Science (EMFISIS) experiment onboard the Van Allen Probes (Kletzing et al., 2013). The data have a temporal resolution of 6 s and a variable uncertainty ranging from 10% to 14%.

**Figure 4:**
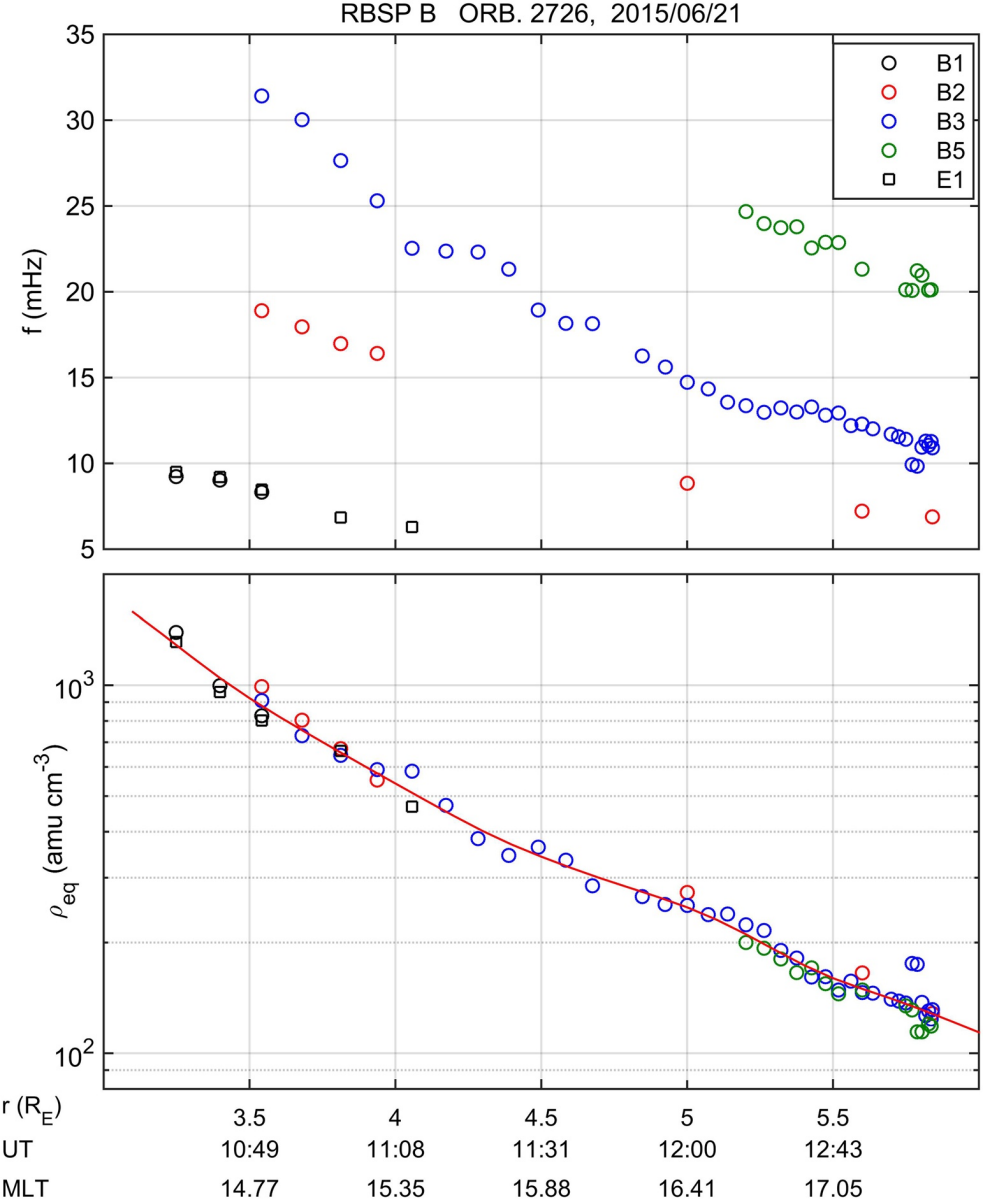
Top panel: Selected harmonic frequencies for the outbound leg of the orbit n. 2,726 of RBSP B on June 21, 2015. The black squares (labeled E1) correspond to the fundamental harmonic detected in *E*_ν_. The circles correspond to the fundamental (B1), second (B2), third (B3), and fifth (B5) harmonic detected in *B*_ϕ_ and are distinguished with different colors as indicated in the legend. Bottom panel: Corresponding estimates of the equatorial plasma mass density. See text for details.

We also used magnetic field measurements made by the fluxgate magnetometer of the EMFISIS experiment, and electric field measurements made by the Electric Field and Waves (EFW) instrument (Wygant et al., 2013) onboard both Van Allen Probes to detect harmonic frequencies of toroidal mode standing Alfvén waves. The detected frequencies were then converted to equatorial or local plasma mass density estimates. When expressed in magnetic field‐aligned (MFA) coordinates, toroidal mode waves are identified in the azimuthal component of the magnetic field (*B*_ϕ_) and in the radial component of the electric field (*E*_ν_). Toroidal frequencies were then determined by first searching for peaks in the *B*_ϕ_ and *E*_ν_ power spectra which were computed in a moving 15 min data window shifted in 5‐min steps. For each significant peak, a weighted average frequency (with the weight given by the corresponding power spectral density) was then computed within a given band around the spectral peak (see Takahashi et al., 2015 for more details). The top panel of Figure 4 shows the harmonic toroidal frequencies which were selected for the outbound leg of the orbit n. 2,726 of RBSP B on June 21, 2015. The black squares (labeled E1) correspond to the fundamental harmonic detected in *E*_ν_. The circles correspond to the fundamental (B1), second (B2), third (B3), and fifth (B5) harmonic detected in *B*_ϕ_ and are distinguished with different colors as indicated in the legend. The bottom panel of Figure 4 shows the corresponding estimates of the equatorial mass density derived from each detected harmonic frequency using the T02 magnetic field model and a radial dependence of the density along the field line ∝ *r* ^−1^. The error associated to each estimate may have different sources (spectral method, poloidal‐toroidal mode coupling, magnetic field model, functional dependence of the density along the field line, etc.,), but we expect the error to be larger for the densities derived from the fundamental harmonic because of the larger relative error in the frequency estimation. In particular, for densities derived from the fundamental harmonic the uncertainty (related to the peak bandwidth) can be as high as ∼50%, while for the densities derived from higher harmonics it is typically of the order of 30%. In any case, the example shows that the densities derived from different harmonics are consistent with each other. The red line is a smoothing spline applied to the experimental points and it is used to evaluate the density at any desired distance.

### 2.3 Plasmapause Test Particle Simulation

In order to provide contextual information for the local measurements, we used a plasmapause test particle (PTP) simulation (Goldstein, De Pascuale, et al., 2014). Starting from an initial configuration specified by the Kp‐based empirical plasmapause model of O'Brien and Moldwin (2003), the model predicts at any next time the global shape of the plasmapause in the equatorial plane using an ensemble of cold test particles subject only to ExB drift. The convection electric field is driven by solar wind data and the Kp geomagnetic index (see Goldstein, De Pascuale, et al., 2014; for further details). The PTP model has demonstrated its validity when compared to global images of the plasmasphere from IMAGE EUV (Goldstein et al., 2005), observations at geostationary orbit (Goldstein, Thomsen, & DeJong, 2014), and Van Allen Probes observations (Goldstein, De Pascuale, et al., 2014). In particular, Goldstein, De Pascuale, et al. (2014) found that the mean difference in plasmapause encounter time between model and Van Allen Probes observations was about 30–40 min, and the mean model‐observations difference in radial location was ∼0.4 R_E_. The output of the model (freely available at http://enarc.space.swri.edu/PTP/) provides the plasmapause location (in *L*‐MLT coordinates) at a 15‐min cadence, also in a movie format. For a direct comparison with the real observations, we generated virtual observations from this model at a given fixed point P_0_ by constructing an index, I_P0‐PP_, in the following way (where *d*_min_ is the distance from P_0_ to the plasmapause):

IP0‐PP = 0: P0 outside the plasmasphere, *d*_min_ > 0.4 R_E_; IP0‐PP = 1: P0 outside the plasmasphere, *d*_min_  ≤ 0.4 R_E_; IP0‐PP = 2: P0 inside the plasmasphere, *d*_min_ ≤ 0.4 R_E_; IP0‐PP = 3: P0 inside the plasmasphere, *d*_min_ > 0.4 R_E_

In the present study (see Section 3.1), we considered the point of coordinates *L* = 4, MLT = 16.

### 2.4 Midnight Plasmapause Location as Derived From Swarm Measurements

Recenty, Heilig and Lühr (2013) found observational evidence for a close relationship between the position of the night side plasmapause and the inner boundary of small‐scale (< 40 km) field‐aligned currents (SSFACs) observed at low‐Earth orbit, that is, the *L*‐shell across which the intensity of SSFACs increases by orders of magnitude. The correlation between the simultaneous variations of the two boundaries was found to be good at all geomagnetic activity levels and the strongest near midnight, while at other MLTs the dayside plasmapause position correlates well with earlier observed position of the near‐midnight SSFAC boundary (Heilig & Lühr, 2018). The observed time lag corresponds to the corotation time from sunrise to the MLT of the dayside plasmapause crossings. While the location of the SSFAC boundary was found very sensitive to the variations in geomagnetic activity, at a given disturbance level the boundary can be well fitted by a circle. Both the center position and the radius of the circle depend on geomagnetic activity. Based on observations of ESA's Swarm satellites, Heilig and Lühr (2018) introduced a simple boundary model. Applying the model to observations made at any MLT, the midnight position of the boundary can be calculated as described in detail by Heilig and Lühr (2018). For this study, we derived a proxy of the midnight plasmapause position based on this approach. From the Swarm‐detected SSFAC boundary positions, we first estimated the midnight boundary position. Then based on the validation results reported by Heilig and Lühr (2018), we subtracted 0.25 R_E_ from all values to account for the average distance between the two boundaries near midnight.

## 3 Comparative Study

### 3.1 Temporal Variation at *L* = 4, MLT = 16

**Figure 5:**
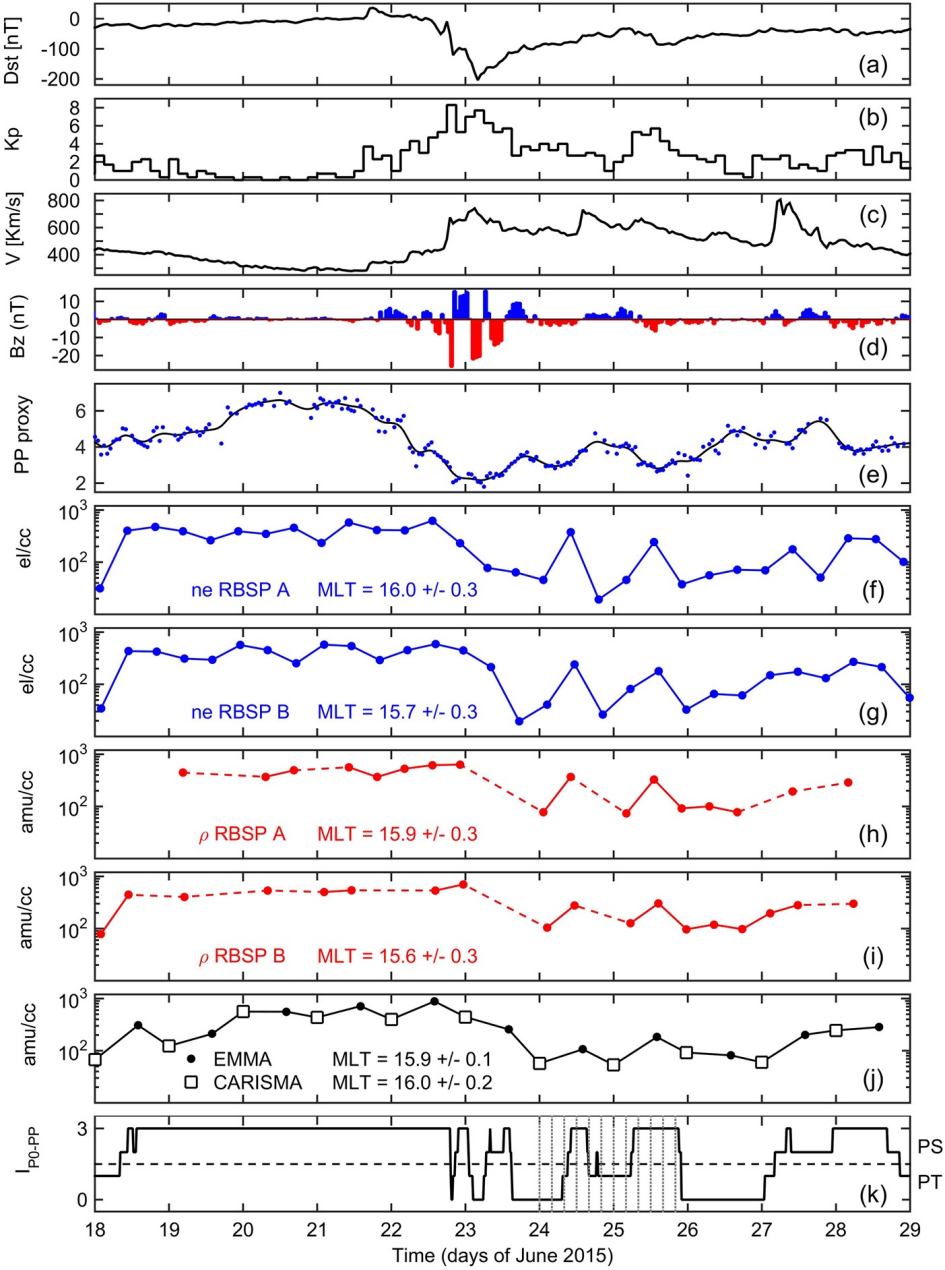
(a–e) Dst, Kp, solar wind speed, B_Z,IMF_ in GSM coordinates (red/blue colors indicate southward/northward direction), and midnight plasmapause proxy during June 18–28, 2015. (f–j) Plasma density evaluated at *L* = 4, 16 MLT. (f, g) RBSP‐NURD electron density. (h, i) Plasma mass density from RBSP‐FLR measurements. (j) Plasma mass density from EMMA/CARISMA‐FLR observations. (k) Virtual observations from the plasmapause test particle simulation in terms of the I_P0‐PP_ index defined in Section 2.3. Values above/below the horizontal dashed line mean that the monitored point is in plasmasphere (PS)/plasmatrough (PT).

We performed a comparison of the temporal variation observed by all of the measurement approaches described above at a fixed location in the magnetosphere during June 18–28, 2015 (Figure 5). Similar studies have been often used in the past using whistler measurements (Park, 1970, 1974), ground FLR measurements (Chi et al., 2000; Dent et al., 2006; Obana et al., 2010), and in situ measurements (Denton et al., 2012, 2016; Reinisch et al., 2004), especially for evaluating long‐term density refilling rates after a depletion event. For the present study, because of the limitations imposed by the Van Allen Probes orbits and FLR measurements, the best point to monitor resulted to be *L* = 4, MLT = 16. The different kind of measurements were obtained as follows.

Equatorial mass density values inferred by EMMA/CARISMA observations (panel j) were obtained by interpolating the corresponding radial density profiles to fixed *r*_eq_ = 4 R_E_ at 14:00 UT/00:00 UT of each day.

Similarly, equatorial mass density values derived from FLRs detected by RBSP A and RBSP B (panels h–i) were obtained by interpolating the radial density profiles to fixed *r*_eq_ = 4 R_E_ (see description of Figure 4).

As regards the electron number density estimates from the NURD plasma wave technique (panels f–g), in order to reduce random measurement fluctuations, the original 6 s data were logarithmically averaged over the interval corresponding to the outbound transit time between the *L*‐shells 3.9 and 4.1 (∼8.5 min). The magnetic latitude of the spacecraft at these passes was always less than 15°, so for the present analysis, no corrections were made to take into account of different distances from the equator from pass to pass. For example, assuming a density variation of *r* ^−1^ along the field line, the density value at a latitude of 15° would be only 7% lower than at the equator.

Lines connecting data points are drawn to guide the eye. They are drawn as dashed lines (panels h–i) when data are missing (no toroidal waves were detected by the spacecraft) between two consecutive observations. For each panel the mean MLT value and the corresponding standard deviation is also indicated.

Also shown in the five uppermost panels (a–e) are the Dst index, the Kp index, the hourly averages of the solar wind speed and of the Z‐component of the IMF in GSM coordinates (solar wind OMNI data), and the hourly averages of the midnight plasmapause proxy as derived from Swarm observations (see Section 2.4). The black line in panel (e) is a smoothing spline through the data. Note the very good correspondence of the midnight plasmasphere erosion phases observed in panel (e) with the intervals of southward direction of B_z,IMF_ (highlighted in red in panel (d)). The greatest erosion occurred at the beginning of June 23 (in correspondence with the Dst minimum) with the midnight plasmapause retreating down to ∼2 R_E_.

As can be seen, the temporal variation is remarkably similar for all density measurements. In particular, the same sequence of decreases and increases through June 23–25 is observed. This pattern could not have been observed at ground if only one latitudinal array (as in Figure 1) had been used. Note also the delay in the afternoon density depletions with respect to the midnight plasmasphere contractions, which is compatible with the time required by the night‐time plasmasphere to corotate into the afternoon sector.

The almost full recovery observed in the middle of June 24 and 25 too quick to be attributed to a refilling from the ionosphere. Indeed, both theoretical arguments (e.g., Rasmussen et al., 1993) and previous experimental observations (Obana et al., 2010; Park, 1974) indicate a duration of several days for an *L* = 4 flux tube to refill after a storm‐associated depletion. A more likely explanation is that an extended plasmasphere structure drifted through the observation point (Denton et al., 2012; Reinisch et al., 2004).

This hypothesis is supported by the virtual observations from the plasmapause test particle (PTP) simulation reported in the bottom panel of Figure 5 in terms of the I_P0‐PP_ index defined in Section 2.3. Values above/below the horizontal dashed line mean that the monitored point is in plasmasphere (PS)/plasmatrough (PT). As can be seen, the virtual observations are qualitatively consistent with the real observations. In particular the index mimics the sequence of the up and down density variations observed during June 24–25. Also worth of note is the correspondence in the strong density increase observed through the first half of June 18.

**Figure 6:**
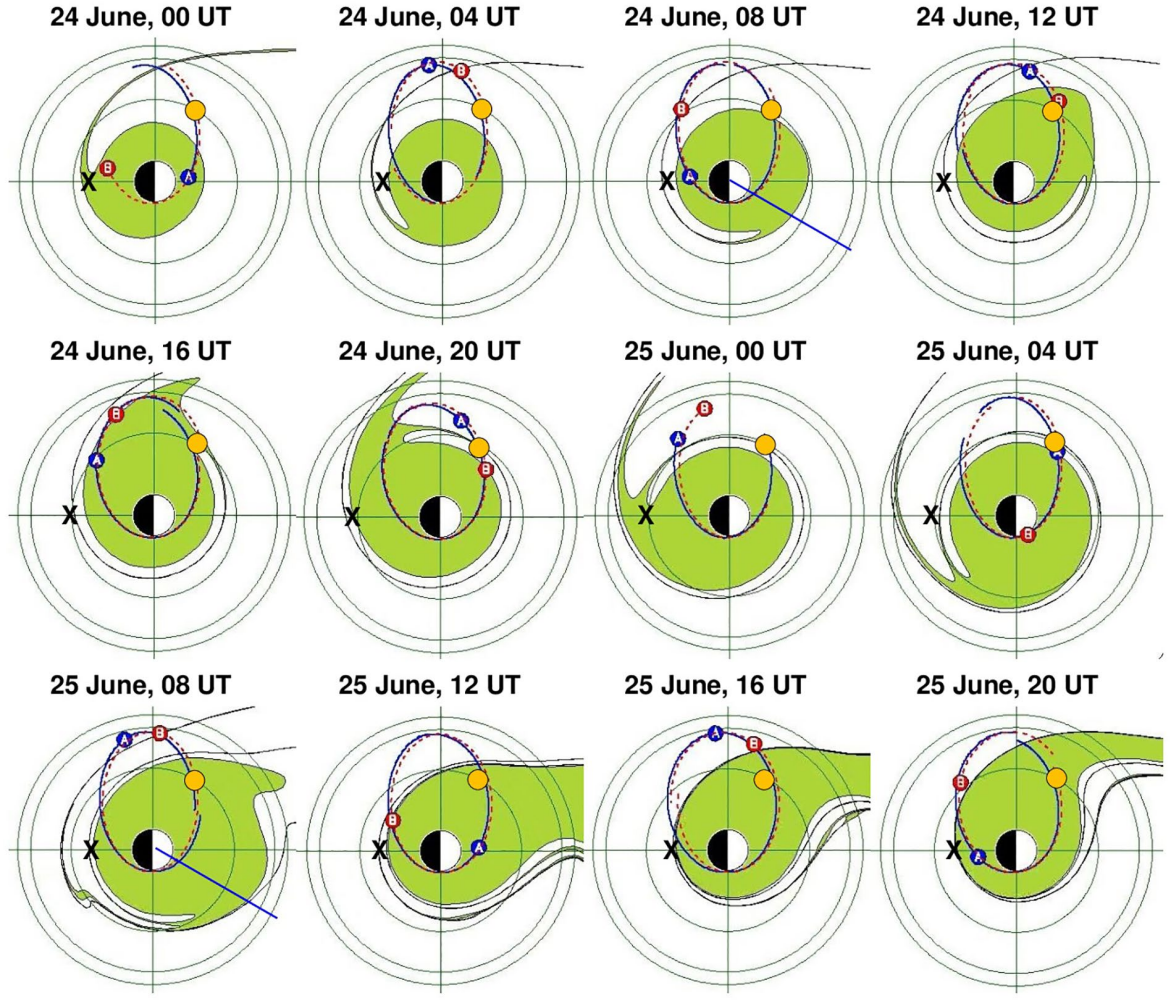
Output of the plasmapause test particle simulation in the equatorial plane (noon is to the right) with a time step of 4 h during June 24–25, 2015. The green regions represent the simulated plasmasphere. Circles are drawn at 4, 6, and 6.6 R_E_. Also shown are the orbits and locations of RBSP A (red) and RBSP B (blue), the monitored point at *L* = 4, 16 MLT (orange dot), and the midnight plasmapause location as determined from Swarm observations (black cross). The blue straight lines drawn at 08 UT of both days indicate 10 MLT.

An overview of the global evolution of the simulated plasmasphere during June 24–25 is shown in Figure 6, with one snapshot every 4 h. The times of these snapshots are marked in Figure 5k with dotted vertical lines. Also indicated in each snapshot are the RBSP A and B locations and orbits, the monitored point at *L* = 4, 16 MLT (orange dot), and the midnight plasmapause location as determined from Swarm observations (black cross). According to the simulation, the density increase observed in the middle of June 24 is interpretable in terms of the rotation of a drainage plume through the monitored point. The increase on the next day (June 25) would be due instead to the sunward surge of the plasma (snapshot at 08 UT) caused by a new enhancement of the convection, as testified by the increase of Kp at 06–09 UT (Figure 5b) and a corresponding southward turning of B_z,IMF_ (Figure 5d). The subsequent plume rotation, when the convection subsided, brought the monitored point to be back outside the plasmasphere at the end of June 25. Note also that for the 10 MLT sector, the strong decrease on June 24 followed by an almost complete recovery on June 25 observed by EMMA (left panels of Figure 1) is consistent with the corresponding snapshots at 08 UT in Figure 6. In fact, on June 24, 08 UT, at 10 MLT (blue straight line) the plasmapause is located at ∼3.4 R_E_, and 24 h later at ∼5.2 R_E_. Also worth of note is the general good agreement of the Swarm‐derived midnight plasmapause location (black cross) with that expected from the PTP simulation.

### 3.2 Conjunction Study

**Figure 7:**
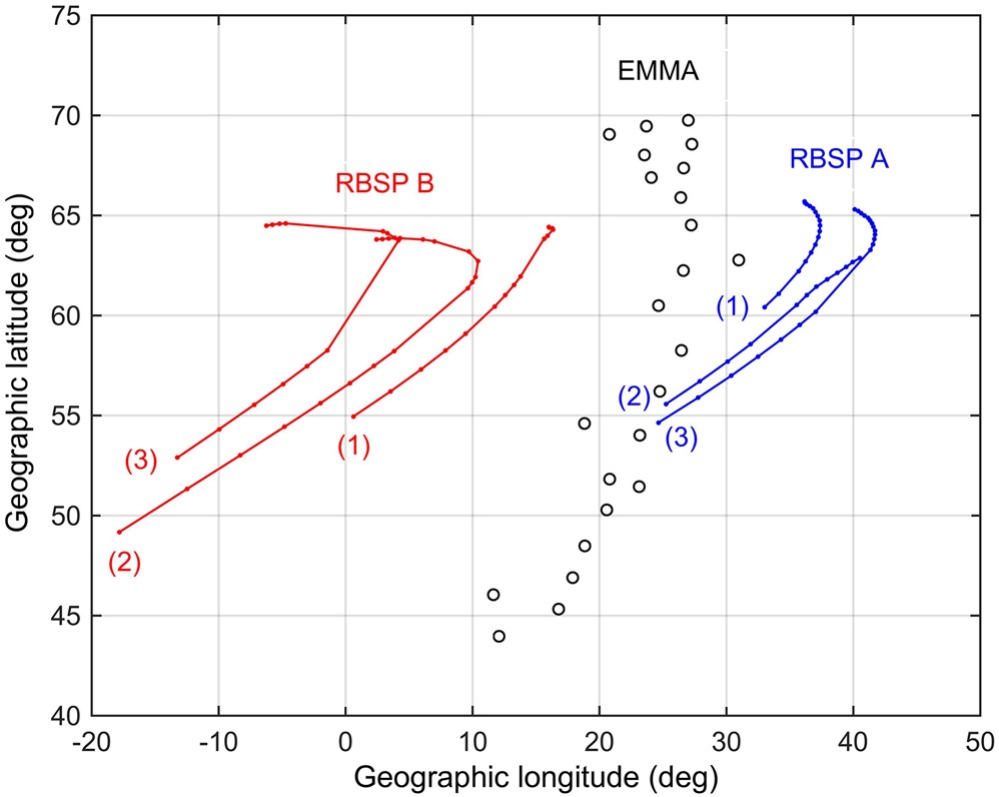
Geographic locations of the EMMA stations and the Radiation Belt Storm Probes (RBSP) magnetic field footprints for six different conjunction intervals (see Table 2). Footprints are determined using the T02 magnetic field model.

We also conducted a more detailed comparison between space and ground measurements by restricting the analysis to the most favorable conjunction periods, that is, to the RBSP orbits for which the magnetic field footprints had the closest approach to the EMMA location. Fairly good conjunctions occurred every eighth RBSP orbit, that is, every third day (on June 22, 25, and 28, 2015). The list of the selected intervals is reported in Table 2, where the start‐end time in the last column is the time interval covered by the RBSP‐FLR measurements. Figure 7 shows the locations of the EMMA stations in geographic coordinates along with the magnetic field footprints of RBSP A and B for the six intervals reported in Table 2. The footprints in Figure 7 (red/blue dots) are evaluated in correspondence of each detected toroidal frequency measurement made every 5 min and are obtained using the T02 magnetic field model.

**Table 2:** Ground‐Space Conjunction Intervals

N	Probe	Orbit n.	Date	DoY	Start‐end time (hh.mm.ss)
1	RBSP‐A	2,744	June 22, 2015	173	12.55.00–14.20.00
2	RBSP‐A	2,752	June 25, 2015	176	12.20.00–13.25.00
3	RBSP‐A	2,760	June 28, 2015	179	12.05.00–14.10.00
1	RBSP‐B	2,729	June 22, 2015	173	13.30.00–15.25.00
2	RBSP‐B	2,737	June 25, 2015	176	13.30.00–17.00.00
3	RBSP‐B	2,745	June 28, 2015	179	13.55.00–17.40.00

**Figure 8:**
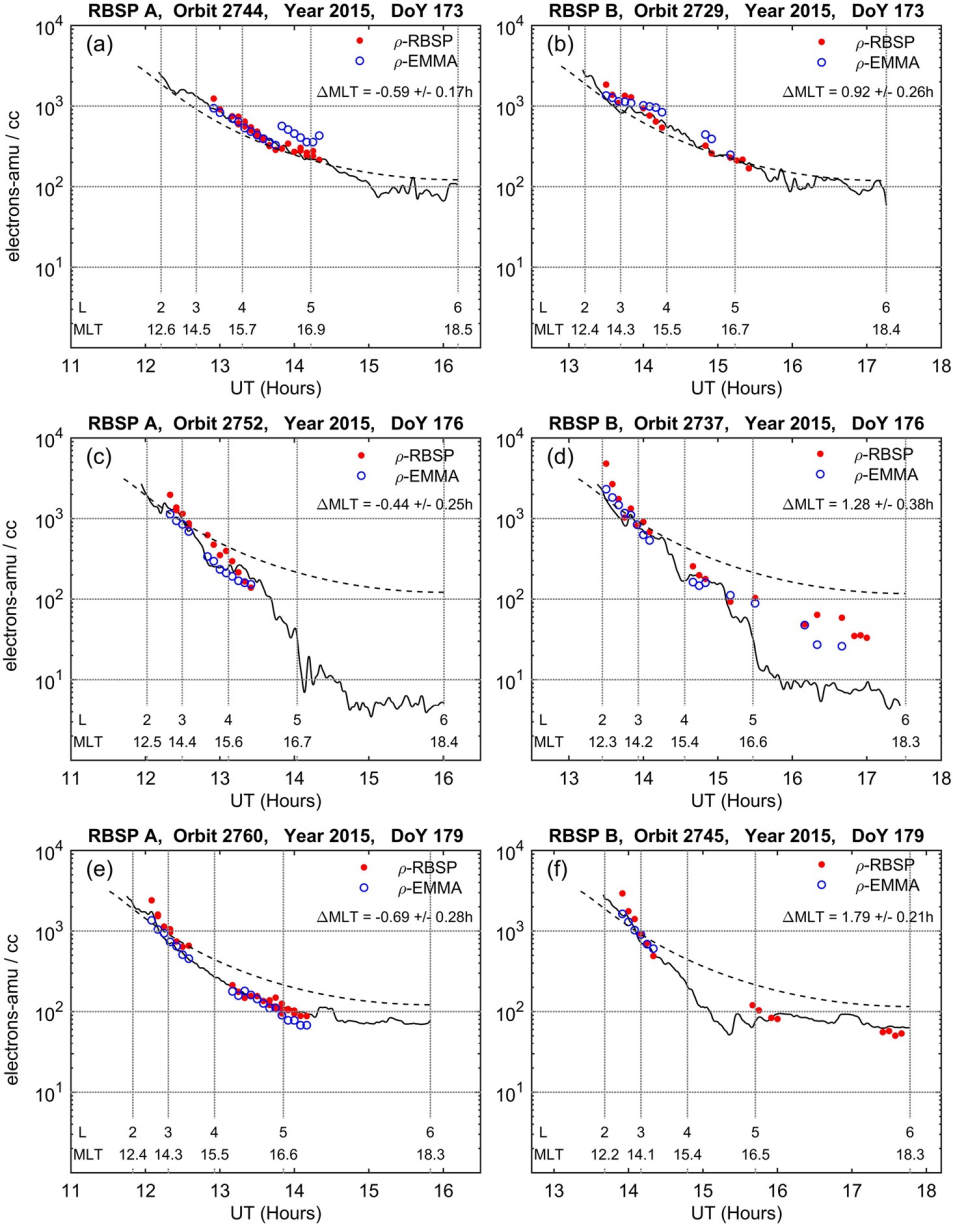
Comparison between RBSP‐equatorial mass densities (red points), EMMA‐equatorial mass densities (blue open circles), and NURD‐equatorial electron densities (black solid line) for the six conjunction intervals listed in Table 2. Dashed curve is the Carpenter and Anderson (1992) saturated plasmasphere electron density model. Dotted vertical lines are drawn at *L* = 2, 3, 4, 5, 6. The mean MLT deviation between EMMA and RBSP measurements is indicated inside each panel. See text for more details.

The results of the ground‐space comparison for each of the selected intervals are shown in Figure 8. The red points are the RBSP equatorial mass densities derived from the toroidal frequencies evaluated at a 5‐min time step. The inversion procedure was applied to all detected harmonics, so different density estimates may be present at a given time. For each RBSP measurement, the closest in time EMMA radial density profile was fitted by a smoothing spline and the fitting value at the RBSP position was taken. These EMMA values are indicated in Figure 8 with blue open circles. The electron density profile (NURD data) is also shown as a black solid line for the entire outbound leg. The original 6‐s local measurements were first converted in equatorial values assuming a radial distribution along the field line ∝ *r* ^−1^ and then a smoothing spline was applied to reduce short‐scale fluctuations due to measurement errors. The dashed curve is the Carpenter and Anderson (1992) saturated plasmasphere electron density model which is drawn as a useful reference. The measurements are plotted as a function of UT, and reference *L* values (and corresponding MLT values) are indicated by dotted vertical lines. The mean MLT deviation (ΔMLT) between EMMA and RBSP measurements is also indicated.

There is a general good agreement between ground and space mass density estimates. Mass density values (in amu cm^−3^) are also generally close to electron number density values (in cm^−3^), which would be consistent with a plasma composed mainly of hydrogen ions. The largest discrepancy is observed in panel (d) where, for *r* > 5 R_E_ (outward of an abrupt density falloff), both RBSP and EMMA mass density values are significantly above the electron number density level, up to a factor of ∼8. This might be indicative of the presence of an oxygen torus just outward of the plasmapause (Fraser et al., 2005).

There is also some indication for the RBSP and EMMA mass density profiles to diverge with decreasing distance in the range 2 < *L* < 3 (see panels (c), (d), (e), (f)), the RBSP estimates being higher than the corresponding EMMA estimates.

**Figure 9:**
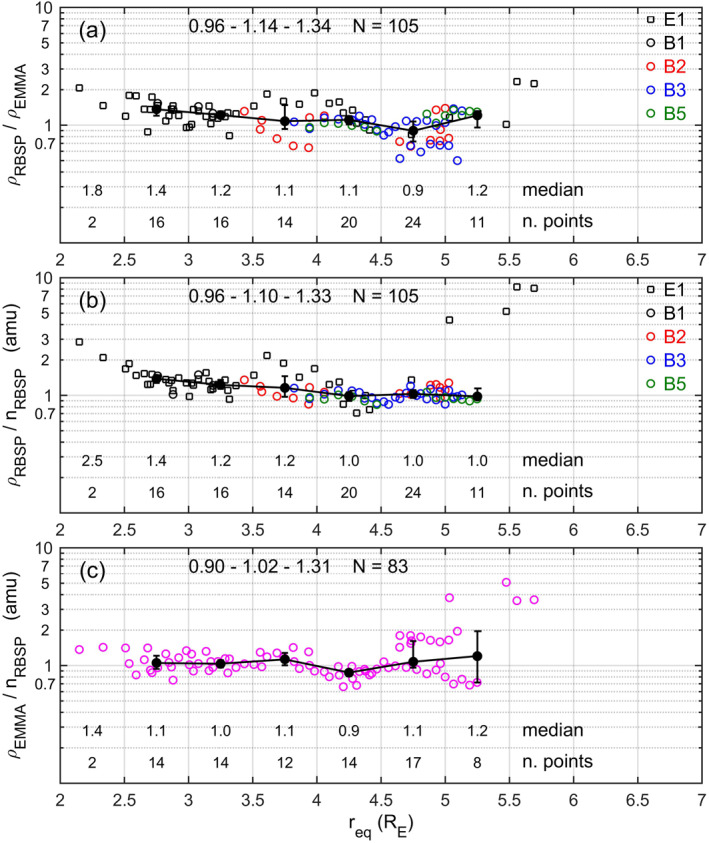
(a) Ratio between RBSP/FLR‐derived and EMMA/FLR‐derived plasma mass density for the conjunction intervals as a function of the equatorial distance. The different markers/colors indicate from which harmonic the Radiation Belt Storm Probes (RBSP) estimate was obtained. Black dots connected by straight lines are the medians in *r*_eq_ bins, and the vertical bars connect the lower and upper quartiles. Lower quartile, median, and upper quartile of the whole population are indicated on the top. Number of samples and medians for each bin are indicated above the horizontal axis. (b) The same as (a) but for the ratio between RBSP/FLR‐derived plasma mass density and RBSP/NURD electron number density (average ion mass). (c) The same as (b) but for the ratio between EMMA/FLR‐derived plasma mass density and NURD electron number density.

The observations for these conjunction events have been statistically analyzed and the results are shown in Figure 9. Panel (a) shows the equatorial density ratio ρ_RBSP_/ρ_EMMA_ as a function of *r*_eq_. 105 sample pairs were available for this analysis. The different markers/colors indicate from which harmonic the RBSP estimate was obtained. The lower quartile (0.96), the median (1.14), and the upper quartile (1.34) of the whole population are indicated on the top. The black dots connected by straight lines are the medians in *r*_eq_ bins, and the vertical bars connect the lower and upper quartiles. The number of samples and medians for each bin are also indicated above the horizontal axis. These results indicate a very good agreement between plasma mass densities derived from ground and in situ FLR observations at all distances, but also confirm systematically higher RBSP estimates for *r*_eq_ < 3 R_E_.

Panel (b) is the plot of the local RBSP mass density over the corresponding electron number density, i.e., the estimated local average ion mass *M*. The RBSP mass densities are the same used in panel (a) but converted to local mass densities at the RBSP position assuming a density variation of *r* ^−1^ along the field. The electron number density values were obtained by taking a log average over 5‐min windows around the central time of the FLR measurements. The results look very similar to those of panel (a). The higher *M* values obtained for *r*_eq_ < 3 R_E_ are then possibly due to an overestimation of the mass density (underestimation of the FLR frequency) from the spacecraft data rather than to a real increase of the average ion mass (see discussion below).

Panel (c) is the plot of the local average ion mass, but using EMMA measurements for the mass density. As expected from the results of panels (a) and (b), the *M* values are slightly lower and closer to 1 amu, even for *r*_eq_ < 3 R_E_.

**Figure 10:**
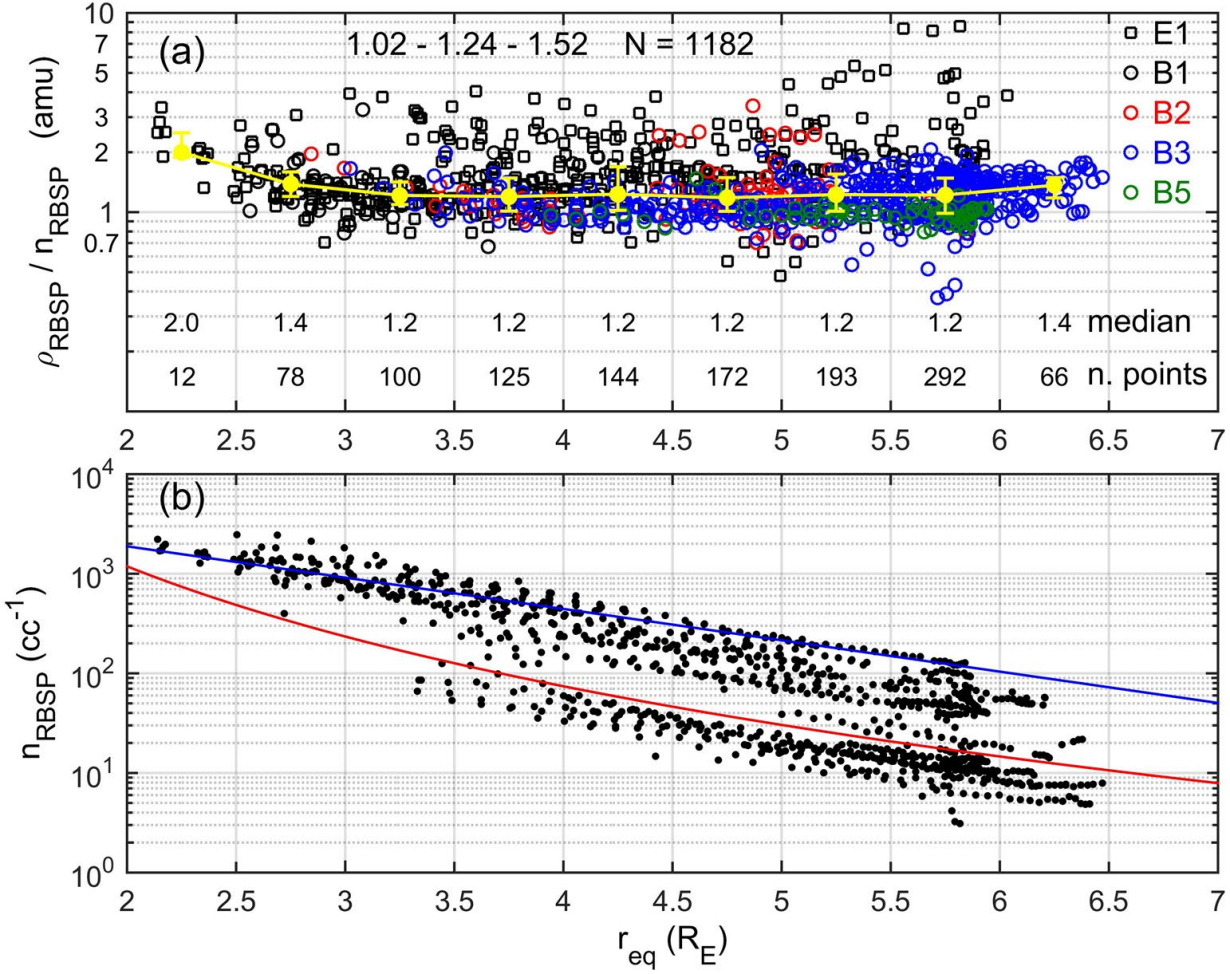
(a) The same as in Figure 9b but for the whole Radiation Belt Storm Probes (RBSP)/field line resonance (FLR) data set. (b) Electron number density (black dots) as a function of the equatorial distance. The red line is the empirical criterion for separating plasmaspheric‐like from trough‐like observations (Sheeley et al., 2001). The blue line is the Carpenter and Anderson (1992) saturated plasmasphere electron density model.

The previous results are restricted to the time intervals with good RBSP‐EMMA conjunction. In Figure 10a the analysis of the average ion mass is extended using the whole RBSP‐FLR data set for June 18–28, 2015. For a better consistency with the previous analysis only outbound passes were considered. 1,182 data points were available, that is, a much larger data set with respect to that used in Figure 9b. The median values are practically identical to those of Figure 9b, except for slightly higher values (∼20%) for *r*_eq_ > 4 R_E_.

A clear increase of *M* for *r*_eq_ < 3 R_E_ is confirmed even for this larger data set. Since we do not find a similar effect when using ground observations (Figure 9c), we argue that it could be due to a downward frequency shift caused by the faster cross‐*L* movement of the spacecraft at lower *L* values. More specifically, during the investigated interval, the RBSP cross‐*L* velocity was maximum at *L* ∼ 1.8. A similar effect was previously found by Anderson et al. (1989), Vellante et al. (2004), Heilig et al. (2013), and Takahashi et al. (2015). The frequency shift was theoretically interpreted either by considering each crossed *L*‐shell oscillating at its own resonance frequency (Anderson et al., 1989), or by considering the satellite movement across the resonance region in case of a monochromatic driving wave (Vellante et al., 2004). In the present study, no RBSP‐FLR measurements were available during inbound passes at *L* < 3, so we could not verify the expected opposite effect, that is, a mass density underestimate due to an upward frequency shift. Another (or additional) possible cause of the higher *M* medians for *r*_eq_ < 3 R_E_ could be a downward bias in the frequency estimate due to the weighted averaging method which was adopted in the frequency selection (Section 2.2). Indeed, due to the typical power law decrease with frequency of the background power spectral density, the frequencies on the left of the spectral peak have on average a larger weight with respect to the frequencies to the right of the peak. This effect should increase with decreasing frequency, and then might be more significant for *r*_eq_ < 3 R_E_ where the fundamental frequency samples are dominant. We evaluated that in some cases the corresponding density overestimation could be up to ∼20%.

An *M* value of 1.2 amu, found in this analysis in the range 3 R_E_ < *r*_eq_ < 6 R_E_, is typical for the plasmasphere (Nosé et al., 2015; Takahashi et al., 2015) which is dominated by H^+^ ions. Larger values (∼3–7 amu) were found, instead, in the plasmatrough by Takahashi et al. (2006, 2008) and Nosé et al. (2011, 2015), using the same technique of the present paper. In order to separate plasmaspheric‐like from trough‐like observations, we used the same empirical criterion adopted by Sheeley et al. (2001), that is, we considered an observation to refer to the plasmasphere/plasmatrough region if the local electron number density was higher/lower than the separation value given by the following expression *n*_o_ = 10 (6.6/*L*)^4^. Figure 10b shows in fact that the electron number density values (black dots) are distributed in two different groups which are quite well separated by the threshold density *n*_o_ (red line). Also shown in the figure is the Carpenter and Anderson (1992) saturated plasmasphere electron density model (blue line). After using this criterion, we found for the plasmatrough a moderate increase in the estimated average ion mass: A median value of 1.55 amu when considering the whole population (398 data pairs) and a maximum median value of 3.0 amu in the 3.0–3.5 R_E_ bin.

Lastly, we examined the effect of using a different power law dependence of the mass density along the field line. All results discussed so far have been obtained by using a power law index *m* = 1 in Equation 1. This choice comes from the results of Vellante and Förster (2006) who, using a plasmaspheric physical‐numerical model, found that the optimal choice for the *L*‐range 2.3–3.4 is *m* ∼ 1 for a large variety of solar and geomagnetic conditions. At higher *L*‐shells, indications on the optimal power law index to use for the mass density distribution along field lines comes from the analysis of the frequency ratios among harmonics of toroidal waves detected on satellites. Takahashi et al. (2004) obtained *m* ∼ 0.5 in the *L*‐range 4–6; Denton et al. (2006) found *m* = 2 appropriate for *L* = 4–5 and *m* = 1 for *L* = 5–6; in a very recent paper, Takahashi and Denton (2021) made separate analysis for the plasmasphere and the plasmatrough in the *L*‐range 4–6 and found *m* = 1.8 in plasmasphere and *m* = 1.9 in plasmatrough. In the same paper, and for the same *L*‐range (4–6), Takahashi and Denton (2021) used a determination of the node latitudes of the detected harmonics of toroidal waves to infer the following appropriate *m* values: 1.4 in plasmasphere and 1.7 in plasmatrough.

We then considered reasonable to assume as a possible range of the power law index: 0 ≤ *m* ≤ 2. For example, Takahashi et al. (2006) and Nosé et al. (2015) used *m* = 0.5. We found no significant differences when using *m* = 0, or *m* = 2 instead of *m* = 1. For example, the median value for the whole population in Figure 10a is 1.24 amu for *m* = 1. This increases to 1.32 amu for *m* = 0, and decreases to 1.16 amu for *m* = 2. The maximum change occurred in the highest *r*_eq_ bin (6–6.5 R_E_) with a variation of ∼ ± 10%. The use of the harmonic frequency ratios to infer the proper power law index has been applied also to ground observations of FLRs by Wharton et al. (2018). They found in plasmatrough at *L* ∼ 5.4 an average *m* value of ∼4. We then repeated the analysis of the average ion mass using also this higher *m* value and found that the median of the inferred *M* values significantly decreased to 0.93 amu when considering the whole *r*_eq_‐range, and to values ∼0.8 amu in the *r*_eq_‐range 4.5–6 R_E_. These values are lower than the minimum physical value of 1 amu, and so the adoption of an *m* value = 4 appears to be less consistent with the expectations.

## 4 Conclusions

The detection of geomagnetic field line resonances by ground‐based magnetometer arrays is a very useful tool for remote sensing temporal and spatial variations of the magnetospheric plasma mass density. For example it has been applied successfully for (a) identifying the plasmapause (Del Corpo et al., 2020; Kale et al., 2007; Menk et al., 2004; Milling et al., 2001), (b) studying the diurnal (Chi et al., 2013; Del Corpo et al., 2019; Waters et al., 1994) and annual (Berube et al., 2003; Menk et al., 2012; Vellante et al., 2007) variations, (c) examining the dependence on the solar EUV irradiance (Vellante et al., 2007), (d) constructing an empirical model in the equatorial plane (Berube et al., 2005; Del Corpo et al., 2020).

The FLR‐technique has been also used to investigate magnetospheric density variations during geomagnetic storms with particular regard to the study of plasmasphere erosion and subsequent refilling from the ionosphere (Chi et al., 2000, 2005; Dent et al., 2006; Grew et al., 2007; Kale et al., 2009; Lichtenberger et al., 2013; Obana et al., 2010; Pezzopane et al., 2019; Piersanti et al., 2017; Villante et al., 2006). However, the use of a single meridional array (which can monitor only one longitudinal sector) does not allow to have a global picture of the spatio‐temporal plasma dynamics during these processes. In addition, without contextual information provided by global observations or models, the causes of the observed variations may not be unambiguously determined.

In the present paper, we found that by combining the observations from two meridional magnetometer arrays (EMMA and CARISMA) longitudinally separated by ∼10 h in local time, we can reproduce the main variations in plasma density observed by the RBSP spacecraft on consecutive passes (every ∼9 h) through the same magnetospheric region (*L* = 4, 16 MLT) during a disturbed period. In addition, the supporting information provided by a plasmapause test particle simulation was crucial to correctly interpret the causes of such variations. In particular, the PTP simulation allowed to interpret rapid recoveries observed after strong density depletions as due to the passage of a drainage plume through the measurement point in one case, and to the sunward surge of the plasma at the beginning of a magnetospheric convection enhancement in another case. So in general, the approach of combining ground remote sensing of mass density using arrays from different longitudes results to be effective for diagnosing the spatio‐temporal mass dynamics associated with the advection of dense plasma which occurs locally on much shorter timescales than plasmaspheric refilling. Obviously more latitudinal chains would be able to reveal more detailed information.

We also conducted a direct comparison between the plasma mass densities derived from ground FLR observations and those derived from space FLR observations for favorable conjunction events. 105 measurements could be compared in the *L*‐range 2–6. To our knowledge, this is the most extensive direct comparison between ground and space FLR measurements. Quite a good agreement was found between the plasma mass densities inferred from the two kind of measurements, with the in situ density estimates being on average 10% higher than the corresponding ground estimates. Larger deviations were found for *L* < ∼3, up to a factor of ∼2 at *L* ∼ 2. This result is qualitatively consistent with a downward shift in the frequency observed by a spacecraft moving outward across the *L* shells, which is expected to increase with decreasing radial distance because of the increasing spacecraft cross‐*L* velocity.

An analysis of the average ion mass using simultaneous RBSP measurements of the mass and electron number density indicates an average ion mass close to 1 amu in the plasmasphere and higher values (typically ∼ 2–3 amu, and up to ∼ 8 amu) in the plasmatrough, consistent with previous observations (Nosé et al., 2011, 2015; Takahashi et al., 2006, 2008).

## Data Availability

Data used in this study are available from the following sources: Zenodo (https://doi.org/10.5281/zenodo.4534059) for EMMA data; NASA/GSFC Space Physics Data Facility Coordinated Data Analysis Web (https://cdaweb.gsfc.nasa.gov) for magnetic and electric field measurements by Van Allen Probes and for OMNI; World Data Center for Geomagnetism, Kyoto (http://wdc.kugi.kyoto-u.ac.jp) for geomagnetic indices; GFZ Data Services: https://dataservices.gfz-potsdam.de/panmetaworks/showshort.php?id=escidoc:5098892 (Zhelavskaya et al., 2020) for NURD electron density data; University of Alberta, Edmonton, Canada (https://www.carisma.ca) for CARISMA data; Space Science and Engineering Division, Southwest Research Institute, San Antonio, Texas (http://enarc.space.swri.edu/PTP) for Plasmapause Test Particle (PTP) Simulations. The SSFAC boundary positions and the plasmapause position proxy used in this study are available as the Midnight Plasmapause Index (PPI) L2 product through the Swarm dissemination portal (https://swarm-diss.eo.esa.int/).

## References

[jgra56551-bib-0001] Anderson, B. J. , Engebretson, M. J. , & Zanetti, L. J. (1989). Distortion effects in spacecraft observations of MHD toroidal standing waves: Theory and observations. Journal of Geophysical Research, 94(A10), 13425–13445. 10.1029/ja094ia10p13425

[jgra56551-bib-0002] Berube, D. , Moldwin, M. B. , & Ahn, M. (2006). Computing magnetospheric mass density from field line resonances in a realistic magnetic field geometry. Journal of Geophysical Research, 111, A08206. 10.1029/2005JA011450

[jgra56551-bib-0003] Berube, D. , Moldwin, M. B. , Fung, S. F. , & Green, J. L. (2005). A plasmaspheric mass density model and constraints on its heavy ion concentration. Journal of Geophysical Research, 110, A04212. 10.1029/2004JA010684

[jgra56551-bib-0004] Berube, D. , Moldwin, M. B. , & Weygand, J. M. (2003). An automated method for the detection of field line resonance frequencies using ground magnetometer techniques. Journal of Geophysical Research, 108, 1348. 10.1029/2002JA009737

[jgra56551-bib-0005] Carpenter, D. L. (1968). Recent research on the magnetospheric plasmapause. Radio Science, 3(7), 719–725. 10.1002/rds196837719

[jgra56551-bib-0006] Carpenter, D. L. , & Anderson, R. R. (1992). An ISEE/whistler model of equatorial electron density in the magnetosphere. Journal of Geophysical Research, 97(A2), 1097–1108. 10.1029/91JA01548

[jgra56551-bib-0007] Carpenter, D. L. , Anderson, R. R. , Bell, T. F. , & Miller, T. R. (1981). A comparison of equatorial electron densities measured by whistlers and by a satellite radio technique. Geophysical Research Letters, 8, 1107–1110. 10.1029/GL008i010p01107

[jgra56551-bib-0008] Chi, P. J. , Engebretson, M. J. , Moldwin, M. B. , Russell, C. T. , Mann, I. R. , Hairston, M. R. , et al. (2013). Sounding of the plasmasphere by Mid‐continent MAgnetoseismic Chain (McMAC) magnetometers. Journal of Geophysical Research: Space Physics, 118, 3077–3086. 10.1002/jgra.50274

[jgra56551-bib-0009] Chi, P. J. , Russell, C. T. , Foster, J. C. , Moldwin, M. B. , Engebretson, M. J. , & Mann, I. R. (2005). Density enhancement in plasmasphere‐ionosphere plasma during the 2003 Halloween Superstorm: Observations along the 330th magnetic meridian in North America. Geophysical Research Letters, 32, L03S07. 10.1029/2004GL021722

[jgra56551-bib-0010] Chi, P. J. , Russell, C. T. , Musman, S. , Peterson, W. K. , Le, G. , Angelopoulos, V. , et al. (2000). Plasmaspheric depletion and refilling associated with the September 25, 1998 magnetic storm observed by ground magnetometers at L = 2. Geophysical Research Letters, 27(5), 633–636. 10.1029/1999GL010722

[jgra56551-bib-0011] Clilverd, M. A. , Menk, F. W. , Milinevski, G. , Sandel, B. R. , Goldstein, J. , Reinisch, B. W. , et al. (2003). In situ and ground‐based intercalibration measurements of plasma density at L = 2.5. Journal of Geophysical Research, 108, 1365. 10.1029/2003ja009866

[jgra56551-bib-0012] Décréau, P. M. E. , Fergeau, P. , Krannosels'kikh, V. , Lévêque, M. , Martin, P. , Randriamboarison, O. , et al. (1997). WHISPER, a resonance sounder and wave analyzer: Performances and perspectives for the cluster mission. Space Science Reviews, 79, 157–193. 10.1023/A:1004931326404

[jgra56551-bib-0013] Del Corpo, A. , Vellante, M. , Heilig, B. , Pietropaolo, E. , Reda, J. , & Lichtenberger, J. (2019). Observing the cold plasma in the Earth's magnetosphere with the EMMA network. Annals of Geophysics, 62(4), GM447. 10.4401/ag-7751

[jgra56551-bib-0014] Del Corpo, A. , Vellante, M. , Heilig, B. , Pietropaolo, E. , Reda, J. , & Lichtenberger, J. (2020). An empirical model for the dayside magnetospheric plasma mass density derived from EMMA magnetometer network observations. Journal of Geophysical Research: Space Physics, 125, e2019JA027381. 10.1029/2019JA027381

[jgra56551-bib-0015] Dent, Z. C. , Mann, I. R. , Goldstein, J. , Menk, F. W. , & Ozeke, L. G. (2006). Plasmaspheric depletion, refilling, and plasmapause dynamics: A coordinated ground‐based and IMAGE satellite study. Journal of Geophysical Research, 111, A03205. 10.1029/2005JA011046

[jgra56551-bib-0016] Dent, Z. C. , Mann, I. R. , Menk, F. W. , Goldstein, J. , Wilford, C. R. , Clilverd, M. A. , & Ozeke, L. G. (2003). A coordinated ground‐based and IMAGE satellite study of quiet‐time plasmaspheric density profiles. Geophysical Research Letters, 30, 1600. 10.1029/2003gl016946

[jgra56551-bib-0017] Denton, R. E. , Jordanova, V. K. , & Fraser, B. J. (2014). Effect of spatial density variation and O+ concentration on the growth and evolution of electromagnetic ion cyclotron waves. Journal of Geophysical Research: Space Physics, 119, 8372–8395. 10.1002/2014JA020384

[jgra56551-bib-0018] Denton, R. E. , Takahashi, K. , Amoh, J. , & Singer, H. J. (2016). Mass density at geostationary orbit and apparent mass refilling. Journal of Geophysical Research: Space Physics, 121, 2962–2975. 10.1002/2015ja022167

[jgra56551-bib-0019] Denton, R. E. , Takahashi, K. , Galkin, I. A. , Nsumei, P. A. , Huang, X. , Reinisch, B. W. , et al. (2006). Distribution of density along magnetospheric field lines. Journal of Geophysical Research, 111, A04213. 10.1029/2005ja011414

[jgra56551-bib-0020] Denton, R. E. , Wang, Y. , Webb, P. A. , Tengdin, P. M. , Goldstein, J. , Redfern, J. A. , & Reinisch, B. W. (2012). Magnetospheric electron density long‐term (*>*1 day) refilling rates inferred from passive radio emissions measured by IMAGE RPI during geomagnetically quiet times. Journal of Geophysical Research, 117, A03221. 10.1029/2011JA017274

[jgra56551-bib-0021] Elkington, S. R. , Hudson, M. K. , & Chan, A. A. (1999). Acceleration of relativistic electrons via drift‐resonant interaction with toroidal‐mode Pc‐5 ULF oscillations. Geophysical Research Letters, 26, 3273–3276. 10.1029/1999GL003659

[jgra56551-bib-0022] Escoubet, C. P. , Pedersen, A. , Schmidt, R. , & Lindqvist, P. A. (1997). Density in the magnetosphere inferred from ISEE 1 spacecraft potential. Journal of Geophysical Research, 102, 17595–17609. 10.1029/97JA00290

[jgra56551-bib-0023] Fraser, B. J. , Horwitz, J. L. , Slavin, J. A. , Dent, Z. C. , & Mann, I. R. (2005). Heavy ion mass loading of the geomagnetic field near the plasmapause and ULF wave implications. Geophysical Research Letters, 32, L04102. 10.1029/2004gl021315

[jgra56551-bib-0024] Goldstein, J. , De Pascuale, S. , Kletzing, C. , Kurth, W. , Genestreti, K. J. , Skoug, R. M. , et al. (2014). Simulation of Van Allen Probes plasmapause encounters. Journal of Geophysical Research: Space Physics, 119, 7464–7484. 10.1002/2014ja020252

[jgra56551-bib-0025] Goldstein, J. , Sandel, B. R. , Forrester, W. T. , & Reiff, P. H. (2003). IMF‐driven plasmasphere erosion of 10 July 2000. Geophysical Research Letters, 30, 1146. 10.1029/2002gl016478

[jgra56551-bib-0026] Goldstein, J. , Sandel, B. R. , Forrester, W. T. , Thomsen, M. F. , & Hairston, M. R. (2005). Global plasmasphere evolution 22–23 April 2001. Journal of Geophysical Research, 110, A12218. 10.1029/2005ja011282

[jgra56551-bib-0027] Goldstein, J. , Thomsen, M. F. , & DeJong, A. (2014). In situ signatures of residual plasmaspheric plumes: Observations and simulation. Journal of Geophysical Research: Space Physics, 119, 4706–4722. 10.1002/2014JA019953

[jgra56551-bib-0028] Grew, R. S. , Menk, F. W. , Clilverd, M. A. , & Sandel, B. R. (2007). Mass and electron densities in the inner magnetosphere during a prolonged disturbed interval. Geophysical Research Letters, 34, L02108. 10.1029/2006gl028254

[jgra56551-bib-0029] Heilig, B. , & Lühr, H. (2013). New plasmapause model derived from CHAMP field‐aligned current signatures. Annales Geophysicae, 31, 529–539. 10.5194/angeo-31-529-2013

[jgra56551-bib-0030] Heilig, B. , & Lühr, H. (2018). Quantifying the relationship between the plasmapause and the inner boundary of small‐scale field‐aligned currents, as deduced from Swarm observations. Annales Geophysicae, 36, 595–607. 10.5194/angeo-36-595-2018

[jgra56551-bib-0031] Heilig, B. , Sutcliffe, P. R. , Ndiitwani, D. C. , & Collier, A. B. (2013). Statistical study of geomagnetic field line resonances observed by CHAMP and on the ground. Journal of Geophysical Research: Space Physics, 118, 1934–1947. 10.1002/jgra.50215

[jgra56551-bib-0032] Horwitz, J. L. , Comfort, R. H. , & Chappell, C. R. (1984). Thermal ion composition measurements of the formation of the new outer plasmasphere and double plasmapause during storm recovery phase. Geophysical Research Letters, 11, 701–704. 10.1029/GL011i008p00701

[jgra56551-bib-0033] Hughes, W. J. , & Southwood, D. J. (1976). The screening of micropulsation signals by the atmosphere and ionosphere. Journal of Geophysical Research, 81(19), 3234–3240. 10.1029/JA081i019p03234

[jgra56551-bib-0034] Jahn, J.‐M. , Goldstein, J. , Kurth, W. S. , Thaller, S. , De Pascuale, S. , Wygant, J. , et al. (2020). Determining plasmaspheric density from the upper hybrid resonance and from the spacecraft potential: How do they compare? Journal of Geophysical Research: Space Physics, 125, e2019JA026860. 10.1029/2019ja026860

[jgra56551-bib-0035] Jakowski, N. , & Hoque, M. M. (2018). A new electron density model of the plasmasphere for operational applications and services. Journal of Space Weather and Space Climate, 8, A16. 10.1051/swsc/2018002

[jgra56551-bib-0036] Kale, Z. C. , Mann, I. R. , Waters, C. L. , Goldstein, J. , Menk, F. W. , & Ozeke, L. G. (2007). Ground magnetometer observation of a cross‐phase reversal at a steep plasmapause. Journal of Geophysical Research, 112, A10222. 10.1029/2007ja012367

[jgra56551-bib-0037] Kale, Z. C. , Mann, I. R. , Waters, C. L. , Vellante, M. , Zhang, T. L. , & Honary, F. (2009). Plasmaspheric dynamics resulting from the Hallowe'en 2003 geomagnetic storms. Journal of Geophysical Research, 114, A08204. 10.1029/2009JA014194

[jgra56551-bib-0038] Kletzing, C. A. , Kurth, W. S. , Acuna, M. , MacDowall, R. J. , Torbert, R. B. , Averkamp, T. , et al. (2013). The Electric and Magnetic Field Instrument Suite and Integrated Science (EMFISIS) on RBSP. Space Science Reviews, 179, 127–181. 10.1007/s11214-013-9993-6 PMC10129970 37123883

[jgra56551-bib-0039] Kurth, W. S. , De Pascuale, S. , Faden, J. B. , Kletzing, C. A. , Hospodarsky, G. B. , Thaller, S. , & Wygant, J. R. (2015). Electron densities inferred from plasma wave spectra obtained by the Waves instrument on Van Allen Probes. Journal of Geophysical Research: Space Physics, 120, 904–914. 10.1002/2014JA020857 26167442 PMC4497465

[jgra56551-bib-0040] Lichtenberger, J. , Clilverd, M. A. , Heilig, B. , Vellante, M. , Manninen, J. , Rodger, C. J. , et al. (2013). The plasmasphere during a space weather event: First results from the PLASMON project. Journal of Space Weather and Space Climate, 3, A23. 10.1051/swsc/2013045

[jgra56551-bib-0041] Maeda, N. , Takasaki, S. , Kawano, H. , Ohtani, S. , Décréau, P. M. E. , Trotignon, J. G. , et al. (2009). Simultaneous observations of the plasma density on the same field line by the CPMN ground magnetometers and the Cluster satellites. Advances in Space Research, 43(2), 265–272. 10.1016/j.asr.2008.04.016

[jgra56551-bib-0042] Mann, I. R. , Milling, D. K. , Rae, I. J. , Ozeke, L. G. , Kale, A. , Kale, Z. C. , et al. (2008). The upgraded CARISMA magnetometer array in the THEMIS era. Space Science Reviews, 141, 413–451. 10.1007/s11214-008-9457-6

[jgra56551-bib-0043] Mauk, B. H. , Fox, N. J. , Kanekal, S. G. , Kessel, R. L. , Sibeck, D. G. , & Ukhorskiy, A. (2013). Science objectives and rationale for the radiation belt storm probes mission. Space Science Reviews, 179, 3–27. 10.1007/s11214-012-9908-y

[jgra56551-bib-0044] Menk, F. W. , Ables, S. T. , Grew, R. S. , Clilverd, M. A. , & Sandel, B. R. (2012). The annual and longitudinal variations in plasmaspheric ion density. Journal of Geophysical Research, 117, A03215. 10.1029/2011JA017071

[jgra56551-bib-0045] Menk, F. W. , Mann, I. R. , Smith, A. J. , Waters, C. L. , Clilverd, M. A. , & Milling, D. K. (2004). Monitoring the plasmapause using geomagnetic field line resonances. Journal of Geophysical Research, 109, A04216. 10.1029/2003JA010097

[jgra56551-bib-0046] Menk, F. W. , & Waters, C. L. (2013). Magnetoseismology: Ground‐Based remote sensing of Earth's magnetosphere. John Wiley & Sons, Inc. 10.1002/9783527652051

[jgra56551-bib-0047] Milling, D. K. , Mann, I. R. , & Menk, F. W. (2001). Diagnosing the plasmapause with a network of closely spaced ground‐based magnetometers. Geophysical Research Letters, 28(1), 115–118. 10.1029/2000GL011935

[jgra56551-bib-0048] Moldwin, M. B. (1997). Outer plasmaspheric plasma properties: What we know from satellite data. Space Science Reviews, 80, 181–198. 10.1023/A:1004921903897

[jgra56551-bib-0049] Nosé, M. , Oimatsu, S. , Keika, K. , Kletzing, C. A. , Kurth, W. S. , De Pascuale, S. , et al. (2015). Formation of the oxygen torus in the inner magnetosphere: Van Allen Probes observations. Journal of Geophysical Research: Space Physics, 120, 1182–1196. 10.1002/2014ja020593

[jgra56551-bib-0050] Nosé, M. , Takahashi, K. , Anderson, R. R. , & Singer, H. J. (2011). Oxygen torus in the deep inner magnetosphere and its contribution to recurrent process of O^+^‐rich ring current formation. Journal of Geophysical Research, 116, A10224. 10.1029/2011JA016651

[jgra56551-bib-0052] Obana, Y. , Menk, F. W. , & Yoshikawa, I. (2010). Plasma refilling rates for L = 2.3−3.8 flux tubes. Journal of Geophysical Research, 115, A03204. 10.1029/2009JA014191

[jgra56551-bib-0051] O'Brien, T. P. , & Moldwin, M. B. (2003). Empirical plasmapause models from magnetic indices. Geophysical Research Letters, 30, 1152. 10.1029/2002gl016007

[jgra56551-bib-0053] Park, C. G. (1970). Whistler observations of the interchange of ionization between the ionosphere and the protonosphere. Journal of Geophysical Research, 75(22), 4249–4260. 10.1029/ja075i022p04249

[jgra56551-bib-0054] Park, C. G. (1972). Methods of determining electron concentrations in the magnetosphere from nose whistlers. Radioscience Laboratory Stanford University. Technical Report 3454‐1.

[jgra56551-bib-0055] Park, C. G. (1974). Some features of plasma distribution in the plasmasphere deduced from Antarctic whistlers. Journal of Geophysical Research, 79(1), 169–173. 10.1029/ja079i001p00169

[jgra56551-bib-0056] Pezzopane, M. , Del Corpo, A. , Piersanti, M. , Cesaroni, C. , Pignalberi, A. , Di Matteo, S. , et al. (2019). On some features characterizing the plasmasphere‐magnetosphere‐ionosphere system during the geomagnetic storm of 27 May 2017. Earth Planets and Space, 71, 77. 10.1186/s40623-019-1056-0 PMC6647577 31402843

[jgra56551-bib-0057] Piersanti, M. , Alberti, T. , Bemporad, A. , Berrilli, F. , Bruno, R. , Capparelli, V. , et al. (2017). Comprehensive analysis of the geoeffective solar event of 21 June 2015: Effects on the magnetosphere, plasmasphere, and ionosphere systems. Solar Physics, 292(11), 169. 10.1007/s11207-017-1186-0

[jgra56551-bib-0058] Rasmussen, C. E. , Guiter, S. M. , & Thomas, S. G. (1993). A two‐dimensional model of the plasmasphere: Refilling time constants. Planetary and Space Science, 41(1), 35–43. 10.1016/0032-0633(93)90015-T

[jgra56551-bib-0059] Reinisch, B. W. , Huang, X. , Song, P. , Green, J. L. , Fung, S. F. , Vasyliunas, V. M. , et al. (2004). Plasmaspheric mass loss and refilling as a result of a magnetic storm. Journal of Geophysical Research, 109, A01202. 10.1029/2003JA009948

[jgra56551-bib-0060] Sandel, B. R. , Broadfoot, A. L. , Curtis, C. C. , King, R. A. , Stone, T. C. , Hill, R. H. , et al. (2000). The extreme ultraviolet imager investigation for the IMAGE mission. Space Science Reviews, 91, 197–242. 10.1023/A:1005263510820

[jgra56551-bib-0061] Sandel, B. R. , Goldstein, J. , Gallagher, D. L. , & Spasojevic, M. (2003). Extreme ultraviolet imager observations of the structure and dynamics of the plasmasphere. Space Science Reviews, 109, 25–46. 10.1023/B:SPAC.0000007511.47727.5b

[jgra56551-bib-0062] Sandhu, J. K. , Yeoman, T. K. , Fear, R. C. , & Dandouras, I. (2016). A statistical study of magnetospheric ion composition along the geomagnetic field using the Cluster spacecraft for *L* values between 5.9 and 9.5. Journal of Geophysical Research: Space Physics, 121, 2194–2208. 10.1002/2015JA022261

[jgra56551-bib-0063] Sheeley, B. W. , Moldwin, M. B. , Rassoul, H. K. , & Anderson, R. R. (2001). An empirical plasmasphere and trough density model: CRRES observations. Journal of Geophysical Research, 106(A11), 25631–25641. 10.1029/2000JA000286

[jgra56551-bib-0064] Shprits, Y. Y. , Subbotin, D. A. , Meredith, N. P. , & Elkington, S. R. (2008). Review of modeling of losses and sources of relativistic electrons in the outer radiation belt. II: Local acceleration and loss. Journal of Atmospheric and Solar‐Terrestrial Physics, 70(14), 1694–1713. 10.1016/j.jastp.2008.06.014

[jgra56551-bib-0065] Singer, H. J. , Southwood, D. J. , Walker, R. J. , & Kivelson, M. G. (1981). Alfvén wave resonances in a realistic magnetospheric magnetic field geometry. Journal of Geophysical Research, 86(A6), 4589–4596. 10.1029/JA086iA06p04589

[jgra56551-bib-0066] Spasojević, M. , Goldstein, J. , Carpenter, D. L. , Inan, U. S. , Sandel, B. R. , Moldwin, M. B. , & Reinisch, B. W. (2003). Global response of the plasmasphere to a geomagnetic disturbance. Journal of Geophysical Research, 108, 1340. 10.1029/2003JA009987

[jgra56551-bib-0067] Takahashi, K. , & Denton, R. E. (2021). Nodal structure of toroidal standing Alfvén Waves and its implication for field line mass density distribution. Journal of Geophysical Research: Space Physics, 126, e2020JA028981. 10.1029/2020ja028981

[jgra56551-bib-0068] Takahashi, K. , Denton, R. E. , Anderson, R. R. , & Hughes, W. J. (2004). Frequencies of standing Alfvén wave harmonics and their implication for plasma mass distribution along geomagnetic field lines: Statistical analysis of CRRES data. Journal of Geophysical Research, 109, A08202. 10.1029/2003ja010345

[jgra56551-bib-0069] Takahashi, K. , Denton, R. E. , Anderson, R. R. , & Hughes, W. J. (2006). Mass density inferred from toroidal wave frequencies and its comparison to electron density. Journal of Geophysical Research, 111, A01201. 10.1029/2005JA011286

[jgra56551-bib-0070] Takahashi, K. , Denton, R. E. , Kurth, W. , Kletzing, C. , Wygant, J. , Bonnell, J. , et al. (2015). Externally driven plasmaspheric ULF waves observed by the Van Allen Probes. Journal of Geophysical Research: Space Physics, 120, 526–552. 10.1002/2014JA020373

[jgra56551-bib-0071] Takahashi, K. , Ohtani, S. , Denton, R. E. , Hughes, W. J. , & Anderson, R. R. (2008). Ion composition in the plasma trough and plasma plume derived from a Combined Release and Radiation Effects Satellite magnetoseismic study. Journal of Geophysical Research, 113, A12203. 10.1029/2008JA013248

[jgra56551-bib-0072] Tsyganenko, N. A. (2002). A model of the near magnetosphere with a dawn‐dusk asymmetry 1. Mathematical structure. Journal of Geophysical Research, 107(A8), 12–21. 10.1029/2001ja000219

[jgra56551-bib-0073] Tsyganenko, N. A. , & Sitnov, M. I. (2005). Modeling the dynamics of the inner magnetosphere during strong geomagnetic storms. Journal of Geophysical Research, 110, A03208. 10.1029/2004JA010798

[jgra56551-bib-0074] Vellante, M. , & Förster, M. (2006). Inference of the magnetospheric plasma mass density from field line resonances: A test using a plasmasphere model. Journal of Geophysical Research, 111, A11204. 10.1029/2005JA011588

[jgra56551-bib-0075] Vellante, M. , Förster, M. , Villante, U. , Zhang, T. L. , & Magnes, W. (2007). Solar activity dependence of geomagnetic field line resonance frequencies at low latitudes. Journal of Geophysical Research, 112, A02205. 10.1029/2006JA011909

[jgra56551-bib-0076] Vellante, M. , Lühr, H. , Zhang, T. L. , Wesztergom, V. , Villante, U. , De Lauretis, M. , et al. (2004). Ground/satellite signatures of field line resonance: A test of theoretical predictions. Journal of Geophysical Research, 109, A06210. 10.1029/2004JA010392

[jgra56551-bib-0077] Villante, U. , Vellante, M. , Francia, P. , De Lauretis, M. , Meloni, A. , Palangio, P. , et al. (2006). ULF fluctuations of the geomagnetic field and ionospheric sounding measurements at low latitudes during the first CAWSES campaign. Annales Geophysicae, 24, 1455–1468. 10.5194/angeo-24-1455-2006

[jgra56551-bib-0078] Waters, C. L. , Menk, F. W. , & Fraser, B. J. (1991). The resonance structure of low latitude Pc3 geomagnetic pulsations. Geophysical Research Letters, 18(12), 2293–2296. 10.1029/91GL02550

[jgra56551-bib-0079] Waters, C. L. , Menk, F. W. , & Fraser, B. J. (1994). Low latitude geomagnetic field line resonance: Experiment and modeling. Journal of Geophysical Research, 99(A9), 17547–17558. 10.1029/94JA00252

[jgra56551-bib-0080] Wharton, S. J. , Wright, D. M. , Yeoman, T. K. , James, M. K. , & Sandhu, J. K. (2018). Cross‐phase determination of ultralow frequency wave harmonic frequencies and their associated plasma mass density distributions. Journal of Geophysical Research: Space Physics, 123, 6231–6250. 10.1029/2018ja025487

[jgra56551-bib-0081] Wharton, S. J. , Wright, D. M. , Yeoman, T. K. , James, M. K. , & Sandhu, J. K. (2019). The variation of resonating magnetospheric field lines with changing geomagnetic and solar wind conditions. Journal of Geophysical Research: Space Physics, 124, 5353–5375. 10.1029/2019JA026848

[jgra56551-bib-0082] Wygant, J. R. , Bonnell, J. W. , Goetz, K. , Ergun, R. E. , Mozer, F. S. , Bale, S. D. , et al. (2013). The electric field and waves instruments on the Radiation Belt Storm Probes mission. Space Science Reviews, 179, 183–220. 10.1007/s11214-013-0013-7

[jgra56551-bib-0083] Zhelavskaya, I. S. , Shprits, Y. Y. , Spasojevic, M. , & Kurth, W. S. (2020). Electron density derived with the Neural‐network‐based upper‐hybrid resonance determination algorithm from the Van Allen Probes EMFISIS measurements. GFZ Data Services. 10.5880/GFZ.2.8.2020.002

[jgra56551-bib-0084] Zhelavskaya, I. S. , Spasojevic, M. , Shprits, Y. Y. , & Kurth, W. S. (2016). Automated determination of electron density from electric field measurements on the Van Allen Probes spacecraft. Journal of Geophysical Research: Space Physics, 121, 4611–4625. 10.1002/2015ja022132

